# Ca^2+^ Regulates the *Drosophila* Stoned-A and Stoned-B Proteins Interaction with the C2B Domain of Synaptotagmin-1

**DOI:** 10.1371/journal.pone.0038822

**Published:** 2012-06-12

**Authors:** Carolina Soekmadji, Clement Angkawidjaja, Leonard E. Kelly

**Affiliations:** 1 Department of Genetics, The University of Melbourne, Parkville, Victoria, Australia; 2 Department of Material and Life Science, Osaka University, Suita, Osaka, Japan; University of Oldenburg, Germany

## Abstract

The dicistronic *Drosophila stoned* gene is involved in exocytosis and/or endocytosis of synaptic vesicles. Mutations in either *stonedA* or *stonedB* cause a severe disruption of neurotransmission in fruit flies. Previous studies have shown that the coiled-coil domain of the Stoned-A and the µ-homology domain of the Stoned-B protein can interact with the C2B domain of Synaptotagmin-1. However, very little is known about the mechanism of interaction between the Stoned proteins and the C2B domain of Synaptotagmin-1. Here we report that these interactions are increased in the presence of Ca^2+^. The Ca^2+^-dependent interaction between the µ-homology domain of Stoned-B and C2B domain of Synaptotagmin-1 is affected by phospholipids. The C-terminal region of the C2B domain, including the tryptophan-containing motif, and the Ca^2+^ binding loop region that modulate the Ca^2+^-dependent oligomerization, regulates the binding of the Stoned-A and Stoned-B proteins to the C2B domain. Stoned-B, but not Stoned-A, interacts with the Ca^2+^-binding loop region of C2B domain. The results indicate that Ca^2+^-induced self-association of the C2B domain regulates the binding of both Stoned-A and Stoned-B proteins to Synaptotagmin-1. The Stoned proteins may regulate sustainable neurotransmission *in vivo* by binding to Ca^2+^-bound Synaptotagmin-1 associated synaptic vesicles.

## Introduction

Synaptotagmin-1 (SYT-1) is primarily associated with Ca^2+^-dependent exocytosis of synaptic vesicles (SV). An integral membrane component of SV, SYT-1 is essential for fast Ca^2+^-dependent release of neurotransmitter in *Drosophila*, *C. elegans*, and mouse [1], [2], [3], [4], [5], [6], [7] Structurally synaptotagmins consist of a short luminal domain, a single transmembrane segment, and two tandem C2 domains (C2A and C2B). These C2 domains form Ca^2+^-dependent complexes with the SNARE proteins and hence trigger exocytosis [8], [9], [10]. Initial studies using *Drosophila* have given insight into the essential function of the C2A and C2B domains of SYT-1 [7]. Flies that lack both these domains (AD4 flies) have an equivalent phenotype to SYT-1 *null* flies, as indicated by the absence of fast neurotransmitter release [7]. The C2B deleted-mutant flies (AD1 flies) show a loss of Ca^2+^ cooperativity for synchronous neurotransmitter release and a defect in endocytosis as indicated by depletion of SV, which suggest that the C2B domain is essential for SV cycling. The AD1 phenotype is thought to be due to the inability of SYT-1 to bind the SNARE complex, and the inability of SYT-1 to oligomerize [7]. As well as being the presumptive Ca^2+^ sensor for neurotransmitter release, SYT-1 has also been implicated in endocytosis of fused SV. The C2B domain of SYT-1 binds a number of important proteins and moieties associated with exo- and endocytosis, such as syntaxin, SNAP-25, μ2 and α-adaptin of AP-2, phospholipids and the Stoned proteins [10], [11], [12], [13], [14], [15], [16], [17]. The C2B domain appears to be the target for induction of endocytosis, and it has been shown that it is recognized by components of the AP-2, and hence clathrin-dependent endocytic machinery. This interaction was originally thought to be Ca^2+^-independent and via the lysine-rich motif [18], [19]. Further study has shown the interaction of AP-2 with SYT-1 to be Ca^2+^ and phosphatidylserine-dependent and to involve the µ2 subunit of AP-2 interacting with the lysine-rich motif of C2B SYT-1 [20].

In *Drosophila*, while this lysine-rich motif may be important for defining the size of recycled synaptic vesicles and in the priming/docking step of vesicle exocytosis [21], [22], it plays no role in their rates of endocytosis [21]. The lysine-rich motif mutant suffers a defect in the priming/docking step of vesicle exocytosis [22], causing a ∼35% reduction in the evoked response, and a two fold increase in spontaneous neurotransmitter release [23]. The corresponding mutation in rat (PC12 cells) has been shown to significantly reduce the extent of SYT-1 internalization (>80%) [24], suggesting that the polyK residues are important for both exocytosis and endocytosis. Studies using a GST tagged C2A-C2B domain of SYT-1, in which the polyK residues are mutated to alanines (A), show abolition of C2B oligomerization [25]. These residues are also shown to be the binding site for the synprint peptide derived from the N and P/Q type Ca^2+^ channel and AP-2 [19], and further shown to specifically bind to α-adaptin and µ2 subunit of AP-2 [15], suggesting that the oligomerization of SYT-1 is important for its function in regulating exo- and endocytosis.

Two other regions of the C2B domain have been implicated in retrieval of SYT-1 from the plasma membrane. It has been shown that a tryptophan containing motif, present in the last β-strand of the C2B domain (WHTL), can act as an internalization signal in chimeric proteins expressed in PC12 cells [24], and the substitution of asparagine residues for two aspartates in one of the Ca^2+^-binding loops of the C2B domain, when present in transgenic *Drosophila*, results in the reduction of SV endocytosis to the equivalent of SYT-1 *null* mutant allele [21]. The WHXL motif in β-strand 8 of C2B is reported to mediate internalization to a similar extent to that seen in the C-terminal tail construct where helix A, β-strand 8 and helix B are deleted [26]. The C-terminus, in a chimeric construct triggered internalization; however, investigation of the C2B structure shows that the W404 residue is only partially exposed [26]. In order to allow internalization, there should be a conformational change in the C2B domain that would allow recognition of the WHXL motif. Whether change in conformation of the C2B domain is caused by interaction with other proteins, such as Stoned proteins, or by other mechanisms, is still unclear [26].

Mutation of the C2B residues Asp416 and Asp418 to asparagines (in D3,4N flies) caused a defect in exocytosis due to the disruption of bound Ca^2+^. The observed evoked transmitter release is decreased by 95% and the apparent Ca^2+^ affinity for synaptic transmission is also reduced [21]. However, these defects in exocytosis may be caused by depletion of recycled vesicles, as this mutation also reduces the rate of SV endocytosis to the equivalent of that of a SYT-1 *null* mutant allele. The slower rate of endocytosis in D3,4N flies, is different from that in SYT-1 *null* flies, as D3,4N flies can be rescued by increasing extracellular Ca^2+^, suggesting that Ca^2+^ coordination is important for regulating the rate of endocytosis [21]. In contrast to the polyK mutation, no significant alteration in vesicle size was observed in D3,4N flies [21].

Much research has focused on the role of the AP-2 complex in SYT-1-induced SV endocytosis. Phospholipids have been shown to interact with SYT-1 [27], [28], and it has been shown that the binding of µ2 of AP-2 with the C2B domain of SYT-1 is mediated by the presence of a phospholipid mixture containing phosphatidylserine (PS) [20]. Ca^2+^-triggered rearrangements between proteins and lipids are believed to cause the opening and dilation of a fusion pore which subsequently leads to the exocytic release of the vesicle contents. Ca^2+^ is postulated to first drive C2 domains membrane interactions, which result in a conformational change that triggers a subsequent C2B-mediated oligomerization step [25]. In *Drosophila*, mutations at the *stoned* locus are known to affect the rates of SV endocytosis and alter the presynaptic distribution of SYT-1, even though the distribution of endocytic proteins such as dynamin, α-adaptin and clathrin, are unaltered [29], [30], [31]. This Ca^2+^-mediated conformational change of SYT-1 may be important in mediating the interaction of the Stoned proteins with SYT-1.

The *Drosophila stoned* locus is dicistronic, encoding two polypeptides, Stoned-A (STNA) and Stoned-B (STNB) [32], [33]. Structurally STNA shows no homology to any protein outside of the Insecta, but contains an N-terminal lysine rich domain followed by a coiled coil region (CCD; coiled coil domain) and a C-terminal domain consisting of four repeats, each of which contains an aspartate-proline-phenylalanine (DPF) motif [33]. This DPF motif has been shown in other proteins to interact with the appendage domain of the α-adaptin subunit of AP-2. The STNB protein contains a C-terminal domain that shows homology to the µ-subunit of AP-2 (µHD; µ-Homology Domain) [32], and an extended N-terminal domain rich in proline and containing a series of asparagine-proline-phenylalanine (NPF) motifs that are known targets for EH domain-containing proteins. We have shown that the N-terminal region of STNB has a high affinity for the *Drosophila* intersectin, DAP-160 [33], [34].

The possible roles of the *Drosophila* STNA and STNB proteins have been primarily studied through their interactions with SYT-1 [17]. The binding of STNB to SV is via SYT-I, and appears to be prior to exocytosis, as the blockade of endocytosis in active synapses, using temperature-sensitive dynamin mutant *shibire*, does not prevent STNB-bound vesicles from being recovered, from, presumably, inactive synapses in these dark adapted flies [17]. In vitro studies have shown that STNB, and its mammalian homologue, stonin-2, binds via the µHD to the C2B domain of SYT-1, while STNA also binds to the C2B domain via its CCD [17], [35]. Ca^2+^ may play a role in this interaction. When *Drosophila* heads are homogenized in the presence of Ca^2+^, STNB and SYT-1 are found almost entirely in the plasma membrane fraction while in the absence of Ca^2+^, both STNB and SYT-1 are found in both plasma membrane and vesicle enriched fractions [17]. This suggests that Ca^2+^ may have an important role in the binding of the Stoned proteins to SYT-1. Exactly what the role might be for the Stoned proteins in SV recycling might be, is unclear, yet they would appear to be essential for normal SV recycling in the fly. We therefore continue with our study of these proteins and especially with their interaction with SYT-I. Here we report our investigations of the effect of Ca^2+^ and phospholipids on the binding of the CCD of STNA and the µHD of STNB to the purified recombinant C2B domain of SYT-1. We have found that Ca^2+^ greatly increases the binding of STNA and STNB to the C2B domain of SYT-1, that these interactions are primarily driven by a Ca^2+^-dependent self-association of the C2B domains of SYT-1 molecules and that the likely binding site for STNB resides within the Ca^2+^-binding loops of the dimeric or multimeric C2B protein domains. We have also observed a negative effect of phospholipids on the *in vitro* binding of STNB to the C2B domain of SYT-I.

## Results

### Interactions between STNA/STNB and the Synaptotagmin-1 C2B domain is Ca^2+^-dependent

**Figure 1:**
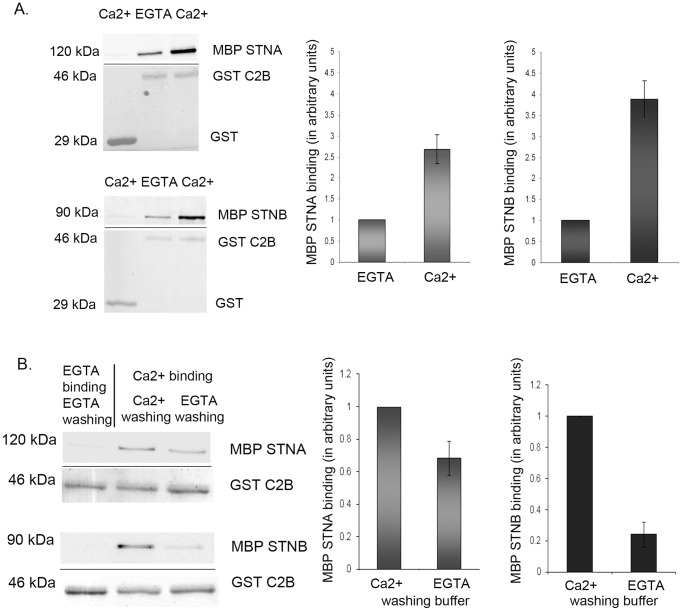
Ca^2+^ enhances the binding of the STNA and STNB proteins to the C2B domain of SYT-1. Preincubation of nucleic acid free GST-C2B with 1mM CaCl_2_ in Hepes binding buffer increased the MBP-STNB (n=6) and MBP-STNA (n=6) binding, compared to preincubation in 1 mM EGTA containing buffer. In control, neither MBP-STNA nor MBP-STNB binds to GST protein in the presence of 1 mM CaCl_2_. The quantified MBP-Stoned proteins are normalized against the amount of eluted GST-C2B. The bound MBP-Stoned proteins in Ca^2+^ are normalized against MBP-Stoned proteins bound in EGTA buffer. Each value represents mean ± S.E.M. **B.** The STNA and STNB proteins, bound to GST-C2B in the presence of Ca^2+^, were subsequently washed with 1 mM EGTA-containing buffer. Washing in EGTA reduced the amount of bound MBP-STNB (n=3) and MBP-STNA (n=3) proteins, compared with equivalent washes in buffer containing 1 mM CaCl_2_ (Ca^2+^ washing buffer). The bar graph represents the amount of quantified MBP-Stoned proteins. The quantified MBP-Stoned proteins are normalized against the amount of eluted GST-C2B. The bound MBP-Stoned proteins in EGTA buffer is normalized against MBP-Stoned proteins bound to GST-C2B in Ca^2+^buffer. Each value represents mean ± S.E.M. The upper panel of each Western Blot images is the MBP-Stoned proteins bound to GST-C2B and detected by anti-MBP antibody while the lower panel is the Ponceau Red staining of GST-C2B of the respective blots as loading control.

Figure 1A shows that the presence of Ca^2+^ considerably enhanced the binding of both MBP-STNB (MHD) and MBP-STNA (CCD) proteins to the GST SYT-1 C2B domain. For STNA this increase was 2.5 fold (n=6, p<0.05) while for STNB it was 4 fold (n=6, p<0.05). The level of binding of the MBP-STNA and MBP-STNB proteins to GST-C2A was slightly higher than to GST alone. A report has shown that the C2A domain is also capable of binding Ca^2+^, even though, due to incomplete Ca^2+^ coordination, the C2A domain has a low intrinsic *in vitro* affinity for Ca^2+^ [36]. A direct comparison of the binding of the MBP-STNA and MBP-STNB proteins to the GST-C2B and C2A domains indicated that the binding to C2B was 3 to 4-fold greater than the binding to the C2A domain (Figure S1, n=4, p<0.05). In fact, the level of binding to the C2A domain is similar to the binding of MBP-Stoned proteins to the GST-C2B domain in Ca^2+^-free buffer (approximately 30% of binding in Ca^2+^ buffer), and might suggest that the observed binding of STNB and SYT-I in the presence of EGTA represents non-specific binding and that Ca^2+^ plays an essential role in the binding of the Stoned proteins to C2B SYT-1. We had previously shown that STNA and STNB both bound preferentially to the C2B rather than the C2A domain of SYT-1 [17].

After being bound in the presence of Ca^2+^, both STNB and STNA can be dissociated from the C2B domain if the complexes are subsequently washed in EGTA-containing buffer (Fig. 1B, n=3, p<0.05). STNB reverts to the level found when binding is carried out in the presence of EGTA. However, STNA binding is reduced but not abolished, suggesting that when bound, the dissociation of STNA from the C2B domain is less sensitive to the absence of Ca^2+^ than STNB. Ca^2+^ was also shown to effect the STNA/STNB interaction with the C2B domain at physiologically relevant Ca^2+^ levels. The interaction for STNA and STNB with C2B SYT-1 is achieved at Ca^2+^ concentration as low as 100–200 µM free Ca^2+^ (data not shown).

### Phospholipids abolish the binding of STNB to C2B SYT-1

**Figure 2:**
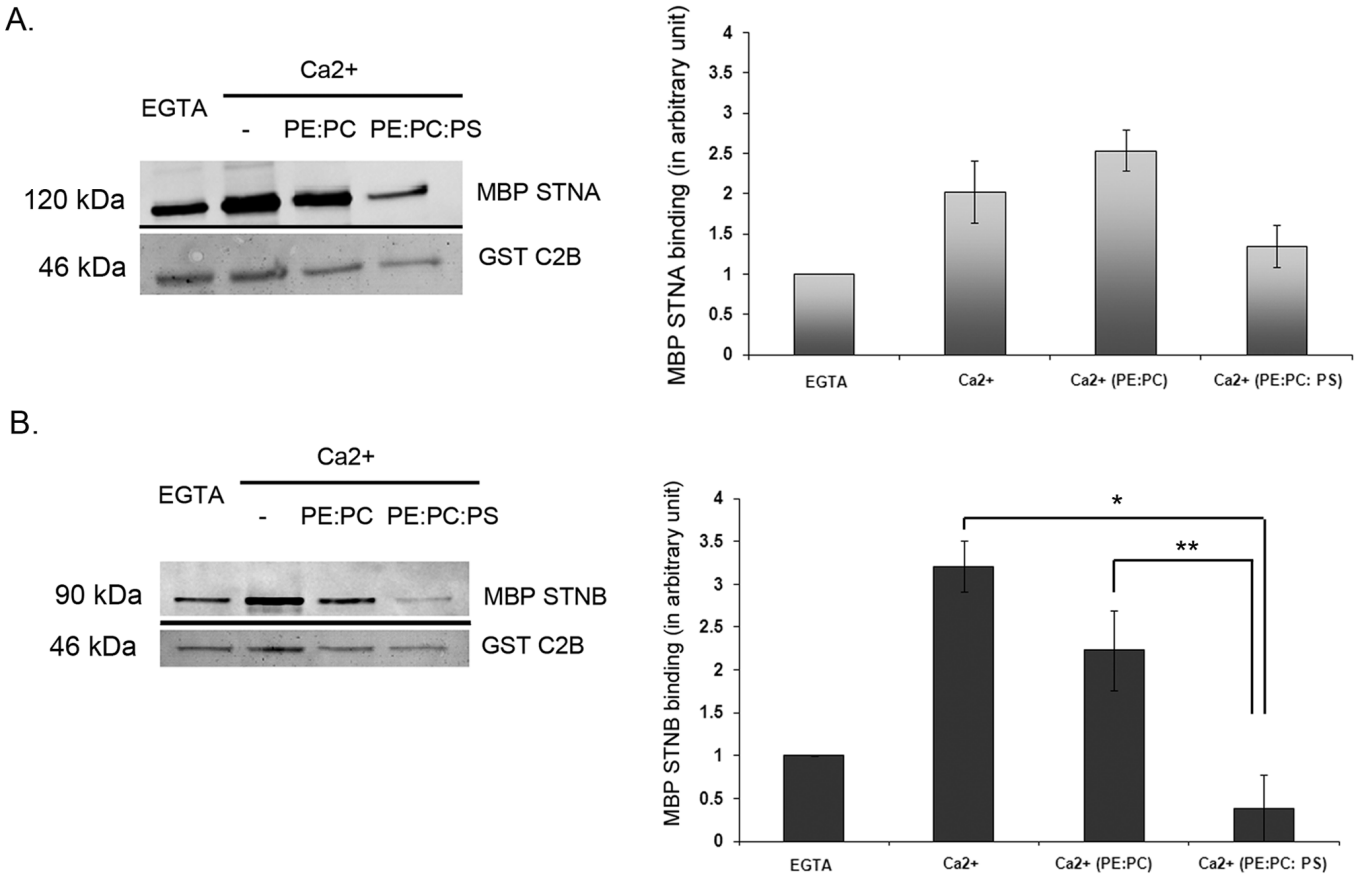
Addition of phospholipids mixture reduces the STNB binding to GST-C2B. Binding of MBP STNA binding to GST-C2B is not affected by phospholipids (n=3). **B.** Phospholipids mixture containing phosphatidylserine (20% PE: phosphatidylethanolamine; 60% PC: phosphatidylcholine, 20% PS: phosphatidylserine) reduces the binding of MBP-STNB (n=4) to GST-C2B in comparison with binding in the presence of Ca2^+^ (*n=4, p<0.01) or PE:PC+ Ca2^+^ (**n=4, p<0.05) to the GST-C2B. “PE:PC” indicates 20%PE:80%PC. “PE:PC:PS” indicates 20%PE:60%PC: 20%PS. The quantified MBP-Stoned proteins are normalized against the amount of eluted GST-C2B. The bound MBP-Stoned proteins are normalized against MBP-Stoned proteins bound to GST-C2B in EGTA buffer. Each value represents mean ± S.E.M. The upper panel of each Western Blot images is the MBP-Stoned proteins bound to GST-C2B and detected by anti-MBP antibody while the lower panel is the Ponceau Red staining of GST-C2B of the respective blots as loading control.

A recent study reported that µ2 cannot replace the function of the µHD of STNB *in vivo* [34], and that the binding conditions of µ2 of AP-2 to the C2B domain require Ca^2+^ and phosphatidyl serine (PS) [20]. We have investigated whether the binding of the µHD of STNB and the CCD of STNA to C2B SYT-1 is also affected by phospholipids such as PS. We have found that the Ca^2+^-dependent binding of MBP-STNB to GST-C2B is completely inhibited by the presence of Triton X-100-produced micelles containing phsopholipid mixtures of phosphatidyl ethanolamine (PE), phosphatidyl choline (PC) and phosphatidyl serine (PS) as compared with the binding in the presence of Ca^2+^ alone and that the critical phospholipid appears to be PS (*p<0.01, n=4; Fig 2A). In the case of STNA, phospholipids also appeared to reduce the binding but this reduction is not statistically significant (n=3; Fig 2B). So, in contrast to the findings with µ2 [20], it would appear that lipids might play a role in inhibiting the interaction between STNB and SYT-I.

### The C-terminal and lysine-rich regions of SYT-1-C2B regulate the binding of STNA and STNB

**Figure 3:**
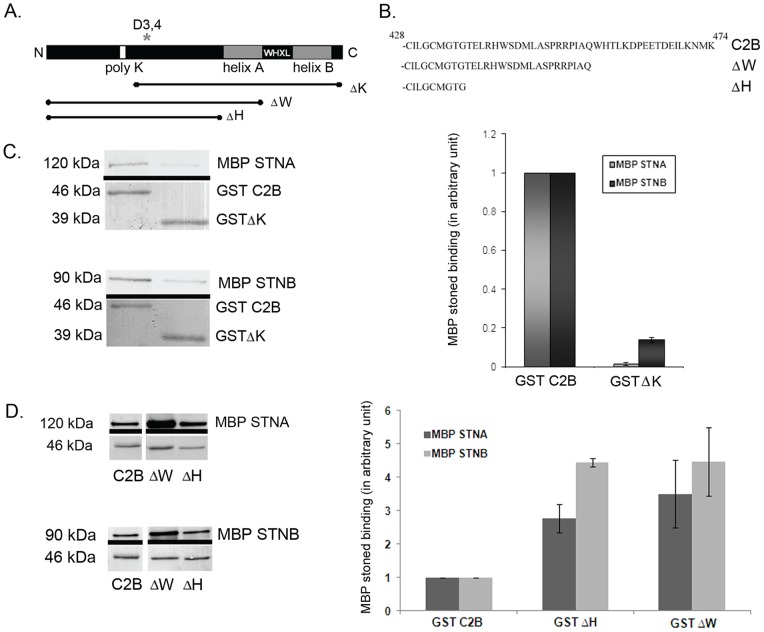
Truncations of the C2B domain of SYT-1 affected STNA and STNB proteins binding. **A.** Several deletion constructs of C2B were created in respect of important residues known to alter the SYT-1 properties. **B.** Amino acid sequence of C-terminal truncated constructs of SYT-1-C2B domain. **C.** Deletion of the N-terminal polylysine region (GST-ΔK) reduced MBP-STNA and MBP-STNB proteins binding, in comparison with binding to GST-C2B. The graph represents the amount of quantified MBP-STNA (n=3) and MBP-STNB (n=3) proteins normalized against the amount of eluted GST-C2B or GST-ΔK fusion proteins in Ca^2+^ buffer. The amount of bound MBP-Stoned proteins to GST-ΔK is normalized against the binding to GST-C2B. Each value represents mean ± S.E.M. **D.** Deletions of C-terminal C2B increased the MBP-STNA and MBP-STNB binding. The graphs represent the amount of quantified MBP-STNA (n=3) and MBP-STNB (n=3) proteins normalized against the amount of eluted GST fusion proteins in Ca^2+^ buffer. The amount of bound MBP-Stoned proteins to GST-ΔH and GST-ΔW is normalized against MBP-Stoned proteins bound to GST-C2B. Experiments were done in Ca^2+^ buffer. Each value represents mean ± S.E.M. The upper panel of each Western Blot images is the MBP-Stoned proteins bound to GST-C2B and detected by anti-MBP antibody while the lower panel is the Ponceau Red stained of GST proteins of the respective blots as loading control.

Various motifs of the C2B domain have been implicated in the recycling of SYT-1 from the plasma membrane. It has been reported that, in PC12 cells, the C-terminal WHTL motif acts as an internalization signal to promote endocytosis of SYT-1 [26]. Another motif, the poly-lysine (polyK) region, is a binding site for the µ2 of AP-2, as well as α-adaptin [15], [19]. To investigate whether any of these endocytic motifs might be responsible for the interaction of C2B with the STNA or STNB proteins, we generated GST-ΔK, which deletes the polyK containing N-terminal region β-strand 1 to β-strand 4 of C2B. We also generated GST tagged C-terminally truncated versions of C2B domain, GST-ΔW and GST-ΔH (Fig. 3). A stop codon was introduced at the loop region, in order to avoid misfolding of the chimeric proteins. The RMSD of the structures during 100 ps MD simulation at 300 K suggested that all the constructs examined have similar structural integrity compared to the full-length C2B (Figure S2). The GST-ΔW construct deletes both the WHTL motif, while GST-ΔH includes a deletion of the helix prior to the β-strand containing the WHTL motif, as well as the terminal helix (Figure 3A and 3B). Both helices are absent from C2A domain.

The binding of both MBP-STNB and MBP-STNA to GST-ΔK is much reduced compared to the wild-type GST-C2B fusion (Figure 3C, n=3, p<0.05). The C2B construct with deletion of the polyK motif has been used to identify α-adaptin, the μ2 subunit of AP-2 and synprint binding [15], [19]. The deletion of the polyK region could possibly alter the folding behavior of the C2B domain of SYT-1 as it deletes half of the C2B domain. However, even though deletion of this region completely abolished STNA binding the deletion did not abolish the MBP-STNB binding (Figure 3C, n=3). In contrast, deletions of the C-terminal region produced a considerable increase in the levels of both STNA and STNB binding (Figure 3D). In the case of STNA, the binding to GST-ΔW increased over threefold (n=3, p<0.05), and for STNB over fivefold (n=4, p<0.05) as compared to the equivalent wild-type construct (Figure 3D). Deletion of both helices and the terminal β-strand (GST-ΔH) also increased the binding of MBP-STNB and MBP-STNA to a similar extent in comparison with the GST-ΔW fusion protein. These data indicate that the helices and the final β-strand, containing the WHTL motif, present in the C-terminal region of the C2B domain do not comprise the binding targets for the Stoned proteins, but must represent an “inhibitory component” of the C2B domain that affects the binding of both STNA and STNB. Perhaps there may be additional factors that interact with the C-terminal region of the C2B domain to regulate the binding of the Stoned proteins to SYT-I.

*In vitro* studies using a GST-C2A-C2B domain, in which the polyK residues are deleted or mutated to alanines, showed an abolition of C2B oligomerization [19], [37]. The C2B oligomerization is thought to regulate vesicle internalization, as alanine substitution of the oligomerization site (polyK to polyA) reduces the extent of internalization of SYT-1 in PC12 cells [24]. The C2A-C2B SYT-1 is also shown unable to oligomerize in the presence of Triton-X 100 treated phospholipids mixture containing PS [25]. As the Stoned binding is reduced by PS containing phospholipids miscelles (Figure 2), these data suggest that the reduction in binding of Stoned proteins might be due to disruption of oligomerization of C2B SYT-1.

**Figure 4:**
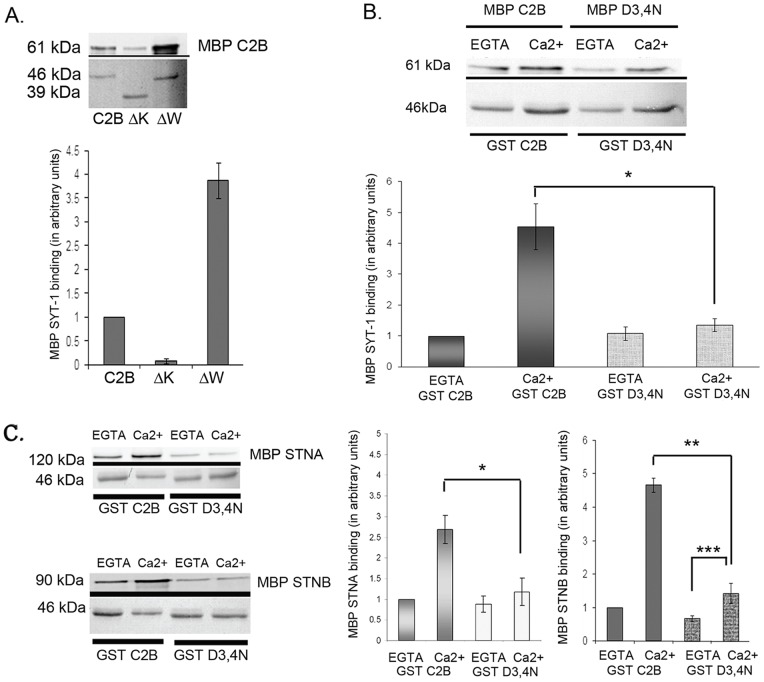
Ca^2^ dependent oligomerization regulates STNA and STNB proteins binding to the C2B domain of SYT-1. **A.** Truncation of C2B affects oligomerization. The N-terminal polylysine deletion (GST-ΔK) reduced MBP-C2B binding while the C-terminal tail deletion (GST-ΔW) increased MBP-C2B binding fourfold (n=3) in comparison with binding to GST-C2B. Independent experiments were done in Ca^2+^ buffer. The amount of quantified MBP proteins is normalized against the amount of eluted GST proteins. The amount of bound MBP-C2B to GST-ΔK or GST-ΔW is normalized against MBP-C2B bound to GST-C2B. Each value represents mean ± S.E.M. **B.** The D3,4N mutation abolished the effect of Ca^2^ to regulate C2B oligomerization in comparison to GST-C2B in Ca^2^ buffer (n=3, p<0.05). The amount of quantified MBP proteins is normalized against the amount of eluted GST proteins. The amount of bound MBP-C2B to GST-C2B or GST-D3,4N is normalized against MBP-C2B bound to GST-C2B in EGTA buffer. Each value represents mean ± S.E.M. **C.** The D3,4N mutation also reduced STNA (*n=3, p<0.05) and STNB (**n=5, p<0.01) binding by comparison with binding to GST-C2B in Ca^2^ buffer. In the presence of Ca^2+^ buffer, the D3,4N mutation significantly increased the MBP-STNB binding in comparison to GST-D3,4N in EGTA buffer (***n=5, p<0.05). The amount of quantified MBP proteins is normalized against the amount of eluted GST proteins. The amount of bound MBP-Stoned proteins to GST-C2B or GST-D3,4N is normalized against MBP-Stoned proteins bound to GST-C2B in EGTA buffer. Each value represents mean ± S.E.M. The upper panel of each Western Blot image shows the MBP tagged proteins bound to GST tagged proteins and detected by anti-MBP antibody; while the lower panel is the Ponceau Red staining of GST proteins of the respective blots as loading control.

We tested whether any of the truncated *Drosophila* C2B constructs affected their Ca^2+^-dependent oligomerization. Unsurprisingly, the wild-type C2B domain was found to self-associate, however this oligomerization was enhanced by Ca^2+^ (Figure 4B, n=3, p<0.05). The truncated GST-C2B fusion proteins (GST-ΔK and GST-ΔW) were also used in self-association experiments with full length MBP-C2B. Consistent with previous findings [37], the GST-ΔK construct did not bind to the wild-type MBP-C2B (Figure 4A, n=3, p<0.01). Under the same reaction conditions however, the GST-ΔW showed an increased capacity to bind MBP-C2B both in the presence Ca^2+^ (fourfold; Figure 4A, n=3, p<0.01) and in EGTA (data not shown). Changes in the level of oligomerization caused by addition of Ca^2+^ or in using the C-terminal truncation constructs, are similar to the changes seen in the binding of the STNA/STNB proteins. It seems likely, therefore, that the increase in binding of MBP-STNA and MBP-STNB to the C2B domain deletions is due to the increase in the ability of these deletions to oligomerize, and that the Ca^2+^ enhancement of STNA and STNB binding to the C2B domain is also due to the Ca^2+^-enhanced oligomerization of the C2B domain.

### Mutation of the Ca^2+^-binding region reduces STNA and STNB binding

If the binding of STNA and STNB to the SYT-I C2B is truly dependent on the Ca^2+^-dependent oligomerization of the SYT-I C2B domain then if the C2B Ca+ binding were to be specifically ablated, then one might expect that this would also lead to a reduction in the binding of both STA and STNB. We therefore asked if the binding of the Stoned proteins is altered in the D3,4N mutant of Syt-I C2B. The D3,4N mutant, replaces two aspartate residues, D416 and D418, with asparagines. Both aspartates are involved in the binding of two Ca^2+^ ions to the C2B domain within the loops [38]. These mutations were reported to reduce the effect of Ca^2+^ on C2A-C2B oligomerization and caused the mutant protein to oligomerize in the absence of Ca^2+^ [37]. The RMSD of the D3,4N structures during 100 ps MD simulation at 300 K suggested that this mutant has similar structural integrity to the full-length C2B (Figure S2).

Using a combination of MBP-D3,4N and GST-D3,4N, we confirm that the D3,4N mutant does abolish the Ca^2+^-enhanced C2B domain self-association (Figure 4B, n=3, p<0.05. As well as reducing the Ca^2+^-dependent self-association of the C2B domain, the D3,4N mutant also reduces both STNA and STNB binding (Figure 4C). This then confirms that the Ca^2+^-induced binding of both STNA and STNB to the SYT-I C2B domain is primarily due to the Ca^2+^-induced self-association of the C2B domain.

### STNB interacts with the Ca^2+^-binding loop of the SYT-1-C2B domain

**Figure 5:**
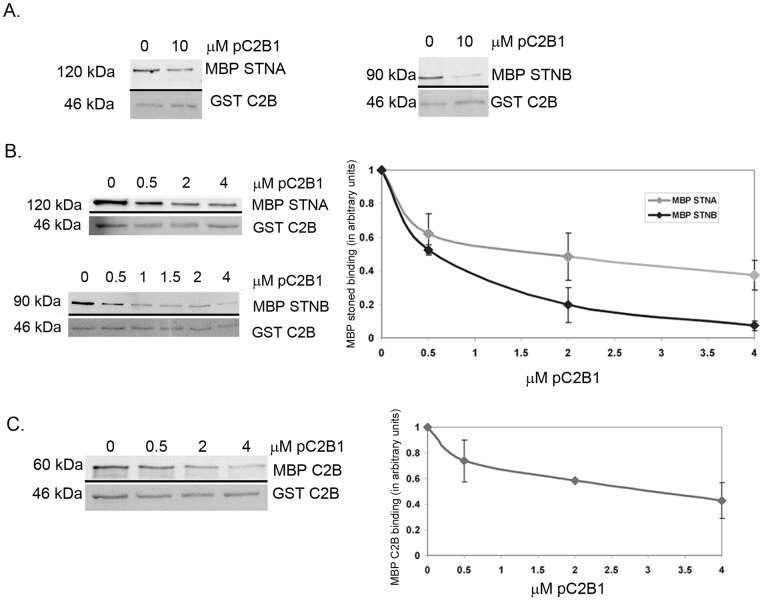
The effect of pC2B1 peptide on STNA and STNB proteins binding and C2B self-association. The experiments were done in the presence of 1mM CaCl_2_. **A.** Addition of 10 µM pC2B1 reduced the MBP-STNA and abolished MBP-STNB binding to GST-C2B domain. **B.** Increasing concentration of pC2B1 in a dose dependent manner (0 to 4 µM) outcompeted MBP-STNB (0.25 µM) binding to 1 µM GST-C2B. The graph represents the amount of quantified MBP-STNA and MBP-STNB proteins normalized against the amount of eluted GST-C2B and the binding of MBP-Stoned in 0 µM pC2B1. Each value represents mean ± S.E.M (n=3). **C.** The pC2B1 peptide also reduced C2B SYT-1 oligomerization. The graph represents the amount of quantified MBP-C2B normalized against the amount of eluted GST- C2B. Each value represents mean ± S.E.M (n=3). The upper panel of each Western Blot image shows the MBP tagged proteins bound to GST-C2B and detected by anti-MBP antibody; while the lower panel is the Ponceau Red staining of GST proteins of the respective blots as loading control.

Since the D3,4N mutant reduces the binding of the STNB protein to the C2B even in the absence of Ca^2+^, we investigated the possibility that the STNB protein might directly interact with this region of the C2B domain. Competition experiments were performed using a peptide, pC2B1. This peptide covers amino acid residues 407–424 and includes all of the residues of loop 3 and β-strand 6 [38], [39]. Addition of the pC2B1 peptide out-competed MBP-STNB in a dose dependent manner (Figure 5A and 5B, n=3). Whereas the peptide also reduced, but did not eliminate, the binding of MBP-STNA to GST-C2B (Figure 5A and 5B, n=3). At 4 µM, pC2B1 abolishes MBP-STNB (0.25 µM) binding to 1 µM GST-C2B. By comparison MBP-STNA binding is reduced but only by 60% (n=3, p<0.05).

When tested for its effects on self-association of the C2B domain, the pC2B1 peptide also reduced C2B oligomerization, but only to an extent similar to the reduction observed for MBP-STNA binding (Figure 5C). At 2 µM, pC2B1 reduces MBP-C2B (0.25 µM) binding to 1 µM GST-C2B by approximately 40%, thus pC2B1 significantly reduces but does not eliminate the self-association of the C2B domain. This would suggest that the partial inhibition of STNA binding was most likely due to the effect of the pC2B1 peptide on oligomerization, while the total inhibition of STNB binding can only be partially due to the failure of the C2B protein to oligomerize in the presence of the pC2B1 peptide. If, however, STNB were to interact directly with residues in the loop, then perhaps the peptide might be competing directly with STNB.

Two other peptides were tested in these assays (Figure S3). The first was a random peptide (pCTRL). This peptide had no effect on the binding of STNB. A second peptide (pC2B1.M) was equivalent to pC2B1 but with the tyrosine at position 417 in the C2B domain substituted with alanine. As tyrosine-containing motifs are known binding sites for the µ-subunit of AP-2, it was hypothesized that such a mutation might alter STNB binding. This Y417A mutant peptide (pC2B.M) behaved in exactly the same way as the wild-type peptide indicating that this tyrosine is not important for STNB binding. Although pC2B1 acts to reduce the oligomerization of the C2B domain, and hence the binding of the Stoned proteins, these data suggest that there is a direct interaction between binding of the loop region of the SYT-1-C2B domain and to the µHD of STNB. Together with the D3,4N binding studies, STNB may bind to the oligomerized C2B domains in the vicinity of the Ca^2+^ binding loop.

## Discussion

In the present study, we have investigated the effect of Ca^2+^ upon Stoned proteins binding to SYT-1 C2B domain. We had reported that the *Drosophila* µHD of STNB binds to C2B domain of SYT-1 [17], which is in agreement with Martina *et* *al.* that showed the µHD of the stonin-2, the mammalian homologue of the *Drosophila* STNB protein, could also bind SYT-1 [35]. Jung *et* *al.* have reported that stonin-2 is able to bind the C2A domain, even though the role of Ca^2+^ in this binding was not explored [40]. We also observed that STNB is able to bind to the C2A domain *in vitro*, albeit at a much lower level as compared to the binding to the C2B domain. Our data show that the binding of *Drosophila* STNB to *Drosophila* SYT-1 is different from that of the murine µ2 subunit of AP-2. While µ2 binding to C2B domain requires PS and Ca^2+^ [20], the binding of the STNA and STNB proteins did not require PS (Figure 2). In fact PS–containing phospholipids nearly abolished the STNB binding to SYT-1 C2B (Figure 2B). Deletion of the polyK region of the C2B domain also did not abolished STNB binding to SYT-1 (Figure 3C), indicating that this region cannot be the binding site for either STNA or STNB. Our data support a recent publication that showed that, in *Drosophila*, µ2 cannot replace the function of the µHD of STNB *in vivo* [41], and suggest that STNB and AP-2 might represent alternative mechanisms for synaptic vesicle recycling.

AP-2 is ubiquitously expressed and implicated in general mechanisms of endocytosis from the plasma membrane, while in *Drosophila*, STNB is expressed and functions only in the nervous system [42]. Differences in the µHD of STNB and the µ2 domain of AP-2 (this study, [20] and [41]) may reflect the need for flexibility of µ2 subunit of AP-2 to act in a number of endocytic pathways, while the function of STNB may be specific for synaptic vesicle retrieval. Our data, in conjunction with a report that STNB may specifically regulate the sorting of a subset of SV [41], suggest the Stoned proteins may regulate sustainable neurotransmission *in vivo* by binding to Ca^2+^-bound SYT-1 associated SV.

Upon Ca^2+^ binding, SYT-1 was reported to undergo a conformational change that protects SYT-1 against trypsin and chymotrypsin digestion *in vitro* [37]. Ca^2+^ has also promoted homo-oligomerization of SYT-1 and/or hetero-oligomerization with other synaptotagmins [37]. Studies using mutations that affect Ca^2+^ dependent oligomerization, such as Y311N, showed a partial inhibition for internalization of SYT-1 in PC12 cells [24] while the corresponding mutation in *Drosophila* (AD3) alters the rate of exocytosis [21]. The AD3 SYT-1 is still capable of binding to SNARE complexes, synprint and AP-2, which implies that the mutation does not cause a complete loss of function [7]. Hence the docked vesicles in AD3 flies require higher Ca^2+^ concentration to undergo exocytosis [7], suggesting that defects in Ca^2+^ dependent oligomerization renders C2B SYT-1 inefficient for exocytosis. It was reported that Ca^2+^-triggered SYT-1 clustering is via the C2B domain and is required for exocytosis [37]. In a potential endocytic model that includes STNB, the oligomerized SYT-1 that has led to exocytosis, could then be a target for STNB binding, and STNB, in turn, could recruit dynamin via the intersectin DAP-160 [34]. This would create what amounts to a Ca^2+^-dependent endocytic complex. However, a previous study has shown that STNB can be found bound to synaptic vesicles, via SYT-1, prior to exocytosis [17], that is, prior to the Ca^2+^ influx that might trigger SYT-1 oligomerization and the coupling of excitation and vesicle fusion [37]. Either this bound STNB reflects the low level of oligomerization of SYT-1 in the absence of Ca^2+^, or that other factors, such as those that might alter the structure of the C-terminal region of SYT-I C2B, are playing a role in potentiating the binding of STNB to SYT-1 even in the absence of Ca^2+^. Certainly there is no STNB protein in the soluble fraction from fly head extracts [17], suggesting that all STNB is in a bound form, and although some is certainly is, perhaps not all is bound to SYT-1.

The D3,4N mutation in transgenic flies results in a reduction in the rate of endocytosis of synaptic vesicles [21], this may be due to a failure of this mutant SYT-I to interact with Stoned proteins. Another mutation which gives constitutive dimerization, the D3,4N mutation [37], was incapable to restore internalization in CHO cells [24]. The D3,4N mutation did not only shows a reduction in the rate of endocytosis in *Drosophila*, but also exocytosis defect by decrease evoked transmitter release and reduce in apparent Ca^2+^ affinity for synaptic transmission [43], [44]. Indeed, similar to these synaptotagmin mutant flies, the *stoned* mutants showed alterations in both spontaneous and evoked release at larval NMJ and severe neurotransmission defect that may lead to embryonic lethality [29], [45], as well as depletion of synaptic vesicle and increase of membrane recycling intermediate that might be due to mislocalization of synaptotagmin during endocytosis [45]. In contrast with µ2 and SYT-1 interaction [20], the binding of Stoned proteins do not require phospholipids. Studies using Folch liposome has shown that D3,4N mutant are not able to induce a close proximity membrane curvature, which may be a prerequisite for SNARE mediated membrane fusion [27]. A synthesized peptide consist of the 3^rd^ loop of C2B SYT-1 can outcompete the µ-homology domain of STNB (our data), suggesting the interaction of STNB to SYT-1 is mediated by a region in loop 3 of C2B domain. Thus, it is an attractive hypothesis that STNB may act as an inhibitor for membrane fusion. In a recent paper [24], it is shown that SYT-1 bound to PtdIns at the same level as PS and that this binding is also required Ca^2+^; while the binding of PIP2 with SYT-1 is less affected by Ca^2+^. It would be interesting to investigate whether these lipids will have similar effect as PS in affecting Stoned and C2B SYT-1 binding.

The role of STNA in the synaptic vesicle cycle remains elusive. The presence of STNA at the larval NMJ appears nonessential [46]. However STNA is certainly associated with synaptic vesicles and it is clear that STNA has a strong affinity for SYT-1 [17]. We have shown that the oligomerization of SYT-1 dramatically increases the binding of STNA and presumably STNA will bind under similar *in vivo* conditions as STNB. The presence of putative AP-2 binding motifs in STNA [29], may make vesicles bound by STNA targets for AP-2 mediated endocytosis. This is in contrast to STNB that lacks such AP-2 binding motifs. Whatever the specific mechanism of action of the STNA protein, it is clear from this study that the action of Ca^2+^ has a marked effect on STNA and STNB association with SYT-1 and confirms them as important proteins in the mechanism(s) of synaptic vesicle recycling in *Drosophila.*

## Materials and Methods

### Recombinant proteins

SYT-1 C2A and C2B constructs were generated in the pGEX-4T-1 vector and expressed as GST-tagged proteins in *E. coli*. The C2A construct (bases 137-326) was produced as described previously [17]. The C2B wild-type construct (bases 323–474) was produced by PCR from the *Drosophila syt* cDNA clone using a 5′ primer that generated an EcoR1 site (5′CTGGTCAGCGT-TGCCTTCGAGGGCGG3′) and the 3′ primer that introduced a terminal Xho1 site as well as an internal BglII site but retained the correct amino acid sequence (5′CTCGA-GTTACTTAATGTTCTTCAAGATCTCGTC3′). The ΔW construct was produced using the same 5′ primer as described above and the 3′ primer (5′CTCG-AGTTACTGGGCGATGGGCCGGCG-3′) that again inserted a terminal XhoI site but also introduced a UAA termination codon that would terminate translation of the C2B domain immediately prior to the WHTL motif. The GST-ΔH was generated by PCR using the same 5′ primer and 3′primer (5′CTCGAGTCCGG-TGCCCATGCAGCCA3′) again inserted a terminal XhoI site but also introduced a UAA termination codon that would delete helix A, helix B and β-strand 8 of the C2B domain. The GST-ΔK was generated using 5′ primer (5′GAATTCTGCACCCTCAACCCTTACTA3′) and 3′ primer (5′CTCGAGTTACTTCAT-GTTCTTCGCGATCTCGTC) to amplify residue C386 to K474. The GST-D3,4N was created by two step PCR amplification using pGEX/C2B as template DNA. The 5′ end was amplified using primer 5′ primer (5′ACCGTCGTGAACTACAACCGTATTGGCAACCTCC3′) and 3′ primer (5′GAGCTGCATGTGTCAGAGG3′). The 3′ end of GST-D3,4N was amplified using 3′ primer (5′GGAGGTGCCAATACGGTTGTAGTTCACGACGGT3′) and 5′ primer (5′CCAGCAAGTATATAGCATG3′). Overlapping PCR was used to generate the full length D3,4N construct using 5′ primer (5′CCAGCAAGTATATAGCATG3′) and 3′ primer (5′GAGCTGCATGTGTCAGAGG3′). The µ-homology domain (µHD) of STNB was amplified using 3′ primer (5′CTCGAGCAGCACCTAGCCAACTTA3′) and 5′ primer (5′GAATTCCGA-GAGCGAGCGTTGACA3′). The DNA sequences encoding the coiled-coil domain (CCD) of STNA and the µHD of STNB were inserted into the pMAL-c2x vector, and expressed in *E. coli* as MBP tagged proteins as previously described [17]. All constructs were verified by sequencing. For the C2B self association assay, the DNA encoding the C2B domain was also inserted into pMAL-c2X, and expressed in *E. coli* to generate MBP-C2B.

### Protein expression and purification

MBP and GST fusion proteins were produced from cultures according to the manufacturers' instructions (NEB and Amersham), and bound to amylose resin (MBP fusions) or glutathione resin (GST fusions). To remove contaminating nucleic acids present in the *E. coli* lysate and known to alter the properties of recombinant C2B, GST-C2B and MBP-C2B fusion proteins bound to resin were washed twice in 10x volume of 20 mM MES pH 6.3, 20 mM CaCl_2_, and 0.6 M NaCl (1h on rotating wheel at 4°C), 10x volume of cold TBS, 10x volume of Tris-CaCl_2_ buffer (1h on rotating wheel at 4°C) and followed by 10x volume of cold TBS, adapted from Ubach et al [47]. The purity of expressed proteins was determined by measuring the A280/260 [20] and analyzed by Coomassie stained SDS PAGE gel.

### Molecular Dynamics simulation

The three dimensional model structures of C2B and its variants (C2B, D3,4N, ΔH and ΔW) were generated using the 3D-JIGSAW comparative modeling server (http://www.bmm.icnet.uk/~3djigsaw/) using the NMR structure of the C2B domain of rat Synaptotagmin-1 (PDB ID 1tjx) as template [38]. Molecular Dynamics (MD) simulation was performed using the program NAMD [48] on a dual-core machine (2.6 GHz) loaded with MS Windows. The CHARMM22 force field was used for the simulation. The input files were prepared using the program VEGA ZZ [49]. The PDB files C2B and its variants were modified by adding hydrogen and solvating in a water box that leaves a 0.2 nm space around the solute. Energy minimization was done for 5000 steps using the steepest descents method. MD simulation began by heating the system from 0 to 300 K for 3 ps, and was followed by an MD simulation at 300K for 100ps timescale using an integration time step of 1 fs. Nonbonded interactions were evaluated with a cutoff distance of 12.0Å and a switch distance of 2.0Å. Langevin dynamics were used during MD simulation, which was done without constraining any degrees of freedom. MD parameters and structures were written every 1ps. The root mean square deviation (RMSD) of the structures during simulation was analyzed using the program VMD [50].

### Protein-protein interaction experiments

Pull down assays were conducted for 2 h at 4°C using 1.5 µM purified GST proteins as bait on 50 µl glutathione resin and 0.25 µM MBP-STNA and MBP-STNB proteins as the prey protein. The pull downs were carried out on a rotating wheel, for 2 h at 4°C, in a total volume of 500 µl of 10 mM HEPES buffer pH 7.4, 1 mM MgCl_2_, 1 mg/ml BSA containing either 1 mM CaCl_2_ (Ca^2+^ binding buffer) or 1mM EGTA (EGTA binding buffer). The beads were then collected by centrifugation, and washed twice with 1ml of binding buffer, followed by 1 ml of cold TBS. The complex of MBP-STNA/STNB and GST-C2B was eluted with 20mM glutathione and analyzed by Western Blot using anti-MBP antibody (New England Biolabs) using Amersham ECL™ Western Blotting System. The amount of MBP-STNA/STNB proteins was then quantified by densitometry using ImageJ or Fuji MacBas software programs and the measured intensity was normalized according to the eluted concentration of GST-SYT proteins visualized by Ponceau S stain. The quantified band density was then normalized to value of binding of respective protein in the presence of EGTA, unless indicated otherwise. For investigating the effect of free Ca^2+^, the GST-C2B was buffered with 1mM EGTA and Ca^2+^ added to yield the indicated free Ca^2+^ concentrations using a p*K*_d_ value of 10.995 for EGTA[51]. Statistical analysis was performed using Microsoft Excel. Significant differences were determined using Student's T-test on two populations of the same variance. P values of less than 0.05 were considered significant.

### Liposome pretreatment of GST-C2B

Liposomes were prepared as described by Zhang *et* *al.* [52]. GST-C2B was preincubated with liposomes (2 mg/ml) for 30 min at room temperature in 20 mM HEPES containing 1 mM CaCl_2_ and then 45 min at 4°C. Composition of liposomes were prepared as described previously, consisted of 60%PC (phosphatidylcholine), 20%PE (phosphatidylethanolamine) and either 20%PS (phosphatidylserine) or a further 20%PC [20]. The purified MBP-Stoned proteins were added and allowed to bind for 2 h at 4°C, followed by washing and analyzed as described above.

### Peptide competition experiments

The peptide pC2B1 (Biotin-KICLVVTVV**D**Y**D**RIGTSE) which contains the aspartate residues (D3,4) at loop 3 of the SYT-1-C2B domain was added at the same time as addition of MBP-tagged Stoned proteins to the GST-C2B domain as described above in the protein-protein interactions assay. Besides pC2B1, pC2B1.M (Biotin-KICLVVTVVD**A**DRIGTSE) and p-CTRL (Biotin-SINPHLGPLQISLGR) were also tested. The binding assays were performed as described above in Ca^2+^ binding buffer.

## Supporting Information

Figure S1 **STNA and STNB proteins bind to the GST-C2B of SYT-1.** The MBP-STNA and MBP-STNB bind significantly stronger to GST-C2B in comparison to GST C2A; 3and 4 fold respectively (n=4, p<0.05). Independent experiments were done in the presence of 1 mM Ca^2+^. The bar graph represents the amount of quantified MBP-Stoned proteins normalized against the amount of eluted GST-C2A or GST-C2B proteins. Each value represents mean ± S.E.M.
(TIF)

Figure S2 **Molecular Dynamics (MD) simulation of C-terminal truncations and D3,4N of C2B.** The RMSD of the structures during 100 ps MD simulation at 300 K suggested all of the C-terminal truncations and D3,4N of C2B examined have similar structural integrity compared to the full-length C2B. The MD simulation data suggested that the deletion/mutation did not affect the protein structure and indicated that all constructs tested are as stable as the *wild type*. Therefore, the changes in biochemical properties reflect the importance of each region deleted or changed by the mutation.
(TIF)

Figure S3 **The effect of pC2B.M peptide on STNB binding.** Increasing concentration of random peptide pCTRL has no effect on MBP-STNB binding to GST-C2B. Similar to pC2B1, addition of pC2B.M reduced the MBP-STNB binding. The experiment was performed in the presence of 1 mM CaCl_2_. The upper panel of Western Blot image shows the MBP-STNB bound to GST-C2B and detected by anti-MBP antibody while the lower panel is the Ponceau Red staining of GST-C2B protein of the respective blots.
(TIF)
