# Supplementary figures and images for: Ca2+ Regulates the Drosophila Stoned-A and Stoned-B Proteins Interaction with the C2B Domain of Synaptotagmin-1
Source: PLoS One. 2012 Jun 12;7(6):e38822. doi: 10.1371/journal.pone.0038822 (PMC3373503; doi:10.1371/journal.pone.0038822)

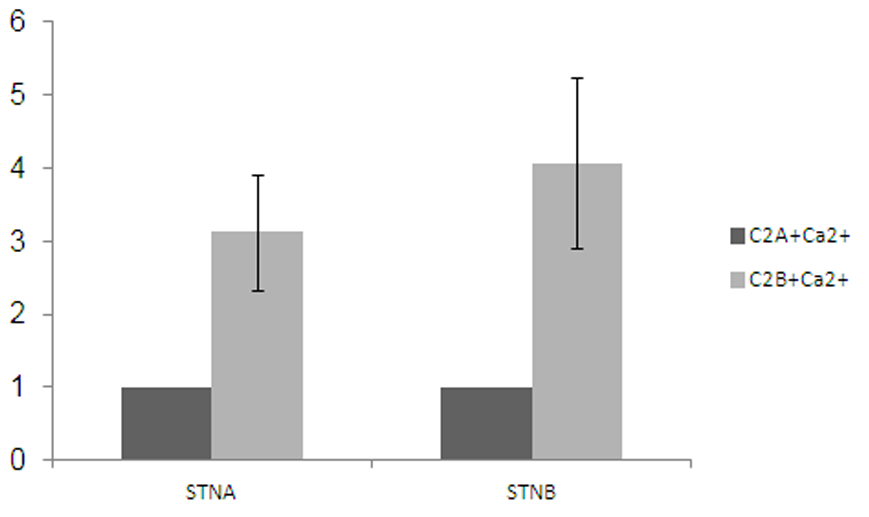

Supplement: Figure S1 — STNA and STNB proteins bind to the GST-C2B of SYT-1. The MBP-STNA and MBP-STNB bind significantly stronger to GST-C2B in comparison to GST C2A; 3and 4 fold respectively (n=4, p<0.05). Independent experiments were done in the presence of 1 mM Ca2+. The bar graph represents the amount of quantified MBP-Stoned proteins normalized against the amount of eluted GST-C2A or GST-C2B proteins. Each value represents mean ± S.E.M. (TIF) [file pone.0038822.s001.tif]

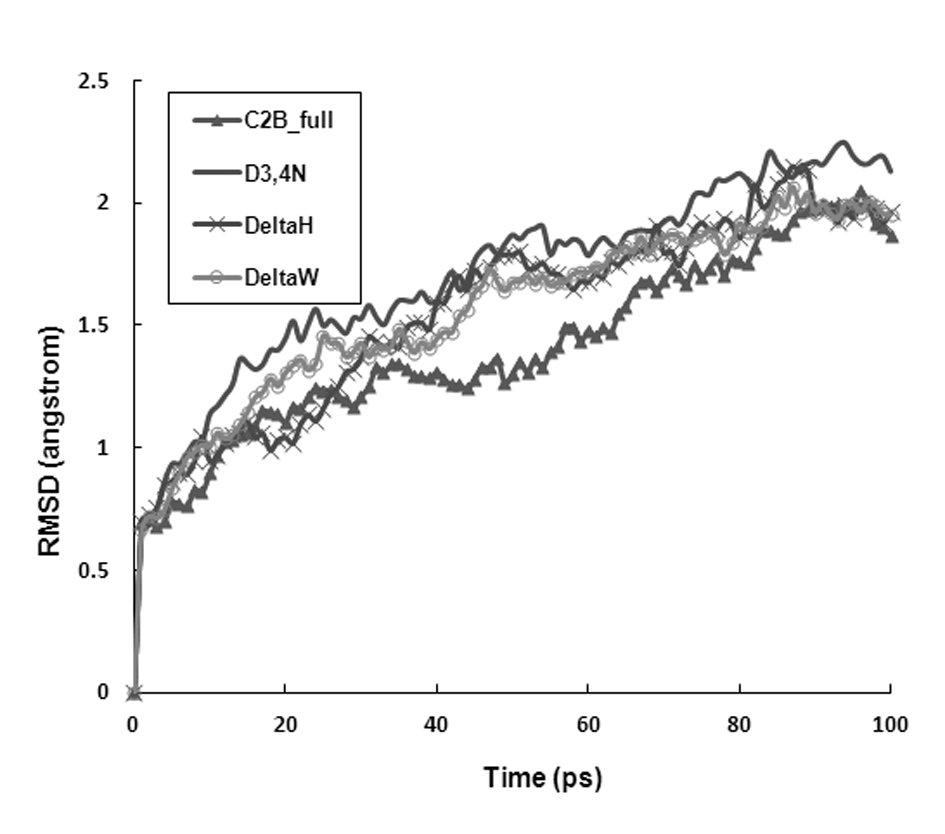

Supplement: Figure S2 — Molecular Dynamics (MD) simulation of C-terminal truncations and D3,4N of C2B. The RMSD of the structures during 100 ps MD simulation at 300 K suggested all of the C-terminal truncations and D3,4N of C2B examined have similar structural integrity compared to the full-length C2B. The MD simulation data suggested that the deletion/mutation did not affect the protein structure and indicated that all constructs tested are as stable as the wild type. Therefore, the changes in biochemical properties reflect the importance of each region deleted or changed by the mutation. (TIF) [file pone.0038822.s002.tif]

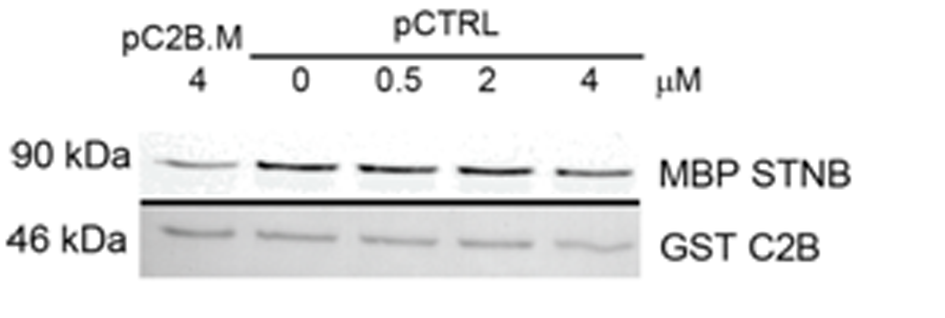

Supplement: Figure S3 — The effect of pC2B.M peptide on STNB binding. Increasing concentration of random peptide pCTRL has no effect on MBP-STNB binding to GST-C2B. Similar to pC2B1, addition of pC2B.M reduced the MBP-STNB binding. The experiment was performed in the presence of 1 mM CaCl2. The upper panel of Western Blot image shows the MBP-STNB bound to GST-C2B and detected by anti-MBP antibody while the lower panel is the Ponceau Red staining of GST-C2B protein of the respective blots. (TIF) [file pone.0038822.s003.tif]
